# *In vitro*reactivation of latent HIV-1 by cytostatic bis(thiosemicarbazonate) gold(III) complexes

**DOI:** 10.1186/s12879-014-0680-3

**Published:** 2014-12-11

**Authors:** Pascaline Fonteh, Debra Meyer

**Affiliations:** Department of Biochemistry, Faculty of Natural and Agricultural Sciences, University of Pretoria, Hatfield Campus, Pretoria, 0002 South Africa

**Keywords:** HIV eradication, Latent virus reactivation, Bis(thiosemicarbazonate) gold(III) complexes, Protein kinase C, TNF-α

## Abstract

**Background:**

A number of cytostatic agents have been investigated for the ability to reactivate latent viral reservoirs, which is a major prerequisite for the eradication of HIV-1 infection. Two cytostatic bis(thiosemicarbazonate) gold(III) complexes (designated **1** and **2**) were tested for this potential in the U1 latency model of HIV-1 infection.

**Methods:**

Cell viability in the presence or absence of **1** and **2** was determined using a tetrazolium dye and evidence of reactivation was assessed by p24 antigen capture following exposure to a latency stimulant, phorbol myristate acetate (PMA) and or test compounds. The latency reactivation mechanism was explored by determining the effect of the complexes on protein kinase C (PKC), histone deacetylases (HDAC) and proinflammatory cytokine production.

**Results:**

The CC_50_ of the complexes in U1 cells were 0.53 ± 0.12 μM for **1** and 1.0 ± 0.4 μM for **2**. In the absence of PMA and at non toxic concentrations of 0.2 and 0.5 μM, **1** and **2** significantly (p ≤ 0.02) reactivated virus in U1 cells by 2.7 and 2.3 fold respectively. In comparison, a 2.6 fold increase (p = 0.03) in viral reactivation was observed for hydroxyurea (HU), which was used as a cytostatic and latent HIV reactivation control. Viral reactivation was absent for the complexes during co-stimulation with PMA indicating the lack of an additive effect between the chemicals as well as an absence of inhibition of PMA induced HIV reactivation but for HU inhibition of the stimulant’s activity was observed (p = 0.01). Complex **1** and **2** activated PKC activity by up to 32% (p < 0.05) but no significant inhibition of HDAC was observed. Increases in TNF-α levels suggested that the reactivation of virus by the complexes may have been due to contributions from the latter and the activation of PKC.

**Conclusion:**

The ethyl group structural difference between **1** and **2** seems to influence bioactivity with lower active concentrations of **1**, suggesting that further structural modifications should improve specificity. The cytostatic effect of **1** and **2** and now HIV reactivation from a U1 latency model is consistent with that of the cytostatic agent, HU. These findings suggest that the complexes have a potential dual (cytostatic and reactivation) role in viral “activation/elimination”.

**Electronic supplementary material:**

The online version of this article (doi:10.1186/s12879-014-0680-3) contains supplementary material, which is available to authorized users.

## Background

Latent reservoirs of HIV result from the integration of viral cDNA into the host cell genome [1]. These latent reservoirs enable viral persistence even in the presence of potent antiretroviral therapy [2]–[4] or immune responses [1]. The latently infected cells are replication competent following reactivation ([5] and thus lead to viral load increases after highly active antiretroviral therapy (HAART) interruption [6],[7]. Reactivation of these reservoirs, while maintaining HAART, is one approach that is being considered for the elimination of latency and HIV infection overall [8]. This approach which has been termed “activation/elimination” aims to flush out latent virus by inducing expression of viral proteins [9]. The high levels of viral protein produced means viral cytopathic effect increases and the virus becomes more visible to the immune system such that cytotoxic CD8+ cells or administered therapeutic agents become more effective.

Various small molecules reactivate latent virus through different mechanisms [10]. Proinflammatory cytokines such as interleukin (IL)-3, IL-6, and TNF-α are one such group of molecules which activate latent infection *in vitro* ([11]. Latent virus reactivation was first reported for PMA in 1988 by Folks and his colleagues [12]. This phorbol ester is also an inducer of monocyte differentiation [13],[14] and of HIV production [15]. The induction of latent virus production by PMA has been associated with the endogenous production of proinflammatory cytokines such as TNF-α [16]. Another phorbol ester, prostratin, also reactivates latent virus and upregulates TNF-α production [17]. Prostratin, like other PKC activators induces virus from latency through the activation of the transcription factor NF-κB [10]. Together with the viral reactivating potential, prostratin also inhibits HIV through the down-regulation of the CD4 coreceptor and has been recommended as a potential inductive adjuvant therapy in HAART because of these combined effects [17]. Another group of compounds that reportedly activate latent virus are HDAC inhibitors. These inhibitors prevent HDAC from producing hypoacetylated nucleosomes at the HIV promoter, hence preventing latency [18]. In addition, cytostatic agents such as actinomycin D and HU reactivate latently infected U1 cells and enhance viral replication [19]. The mechanism of viral reactivation by HU is not clear while actinomycin D has been reported to reactivate virus by modulating cytokine production causing increases in IL-6 and decreases in TNF-β [20]. HU is also known to have anti-HIV effects as a result of its ability to deplete intracellular pools of deoxynucleotide triphosphates (dNTPs) which in turn inhibits viral DNA synthesis making it a cytostatic agent [21]–[23]. The viral reactivating effect of HU in conjunction with its HIV inhibiting ability suggests that this chemical could also be part of an inductive adjuvant therapy with HAART just like prostratin.

We previously reported the antiviral effect of two bis(thiosemicarbazonate) gold(III) complexes (**1** and **2**) and proposed that this effect was as a result of the cytostatic abilities of these complexes [24]. Cytostatic complexes in combination with directly antiviral drugs are proposed as an alternative anti-viral treatment approach referred to as virostatic cocktails [25],[26]. Virostatic combinations involving HU and didanosine have been shown to be clinically effective [27]–[29] but toxicity concerns continue to prevent wider usage. Because some cytostatic agents also reactivate latent virus (HU and actinomycin D as examples) the possibility that the cytostatic complexes **1** and **2**, could also exert an effect on viral reactivation existed and was explored here.

For this study, a promonocytic cell line (U1) which is an *in vitro* model of postintegration latency [19] was used. Postintegration latency unlike preintegration latency occurs when the genome of a provirus is silenced such that effective expression after integration into the host cell genome is prevented [8]. PMA was used as a stimulant for latent virus activation due to its effect on the binding of NF-κB dependent transcription [30],[31], resulting in the transcription of viral genes. The complexes reactivated latent virus at non toxic concentrations. This reactivation appeared to be dependent on PKC activation and to an extent on the endogenous production of TNF-α. The complexes used in this study could form part of virostatic cocktails and the concurrent reactivation of latent HIV suggests that these complexes could potentially be used in virus “activation/elimination” as a means of flushing out latent HIV.

## Methods

### Compounds and gold complexes

**Figure 1:**
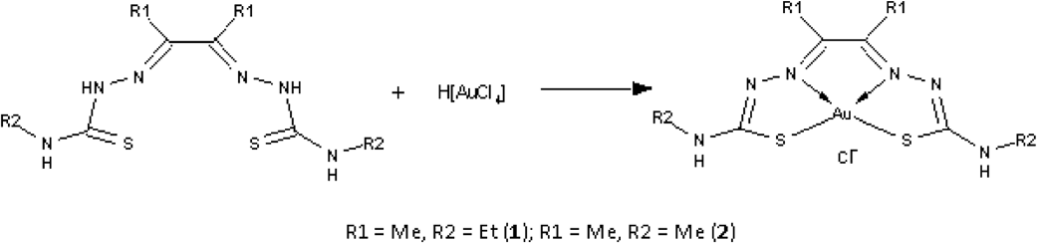
**Molecular structure of complexes 1 and 2 [** [24]**.** This figure is reproduced with permission from the Journal of Inorganic Biochemistry.

The synthesis of the bis(thiosemicarbazonate) gold(III) complexes (**1** and **2**) have previously been reported [24]. The gold(III) complexes have general formula; [Au(L)]Cl, where L = **L1**, diacetyl-bis(N^4^-ethylthiosemicarbazone, **L2**, diacetyl-bis(N^4^-methylthiosemicarbazone). **L1** and **L2** are also known as the precursor compounds or ligands used in synthesising **1** and **2** while HAuCl_4_.4H_2_O is the gold starting material. Complex **1** differs from **2** by the presence of an ethyl group in place of a methyl group as shown in the molecular formulae in Figure 1. HU was purchased from Sigma Aldrich (Missouri, USA). The compounds and complexes were dissolved in DMSO (vehicle) to 20 mg/mL and stored in single use aliquots at −20°C before further dilution with culture media as needed in the various assays to final DMSO concentration of ≤0.6% (v/v).

### Cells

The promonocytic U1 cell line [11] was obtained from the AIDS Research and Reference Reagent Program, NIAID, National Institute of Health (Rockville, MD). U1 cells are a subclone of U937 cells which are chronically infected with HIV-1. CD4 levels are low and constitutive expression of virus is minimal. The cells therefore require the use of stimulants such as the proinflammatory cytokines of the IL family (IL-3, IL-6), TNF-α and granulocyte colony-stimulating factor or PMA to stimulate virus expression [11],[12]. Generally, HIV replication from cell lines and cells from HIV infected people require activation [32]. U1 cells were maintained at 1x10^5^ cells/mL in RPM1 1640 containing 2 mM L glutamine (Sigma Aldrich, Missouri USA), supplemented with 10% fetal bovine serum (Thermo Scientific, HyClone®, UT, USA), 100 U/mL penicillin and 100 μg/mL streptomycin (Thermo Scientific, HyClone®, UT, USA). The cells were subcultured every two days.

### Effect of the complexes on the viability of U1 cells using MTT

U1 cells (5x10^4^ cells/mL) were treated with eight different concentrations of **L1**, **L2**, **1**, **2** as well as HAuCl_4_.4H_2_O and HU ranging from 0.2-100 μM. An untreated vehicle control sample contained cells and 0.6% DMSO, a cell only control, and a blank control contained 0.6% DMSO only. The cells were incubated for 72 h (37°C, 90% humidity, 5% CO_2_) after which 140 μL of spent medium was discarded and replaced with an equivalent amount of complete media and 20 μL of 5 mg/mL MTT. The developed colour was analysed after 2 h following solubilisation of the MTT formazan product using acidified isopropanol. Absorbance was measured at 550 nm and a reference wavelength of 690 nm on a Multiskan Ascent® spectrophotometer (Labsystems, Helsinki, Finland). Cell viability was calculated using the formula: (absorbance of sample - absorbance of medium/ absorbance of vehicle control - absorbance of medium) x 100. The vehicle (0.6%) control did not alter cell viability and was similar to the cell only control. The CC_50_ of the complexes and ligands were graphically obtained after generating a dose response curve using Graphpad Prism® software (California, USA). An ordinary two way analysis of variance (ANOVA) followed by a Tukey’s multiple comparison test was used in determining differences between treatments and p < 0.05 was considered significant.

### HIV-1 replication

In determining complex effect on HIV-1 replication, two approaches were used. The first approach involved determining the effect of the complexes on latent virus reactivation in the absence of stimulant and the second in the presence of stimulant mediated reactivation using PMA (Sigma Aldrich (Missouri, USA). In both approaches, latently infected U1 cells at 5 x10^4^ cells/mL were treated with non toxic concentrations of **L1**, **L2**, **1** and **2** for 72 h (37°C, 5% CO_2_). In the co-stimulation study where complex effect on PMA mediated reactivation of HIV was sought, 3 nM of PMA was added to the test samples 6 h post treatment. PMA also served as a control for reactivation. After the 72 h incubation, cell free supernatant was collected and tested for p24 antigen levels using the RETROtek p24 kit (ZeptoMetrix Corporation, New York, USA) following the manufacturer’s instructions.

### Viral reactivating mechanism

#### Effect of the complexes on HDAC and PKC activity

To determine the mechanism of viral reactivation, complex effect on HDAC inhibition and PKC activation was investigated. A fluorometric HDAC assay kit (Sigma, MO, USA) was used and the assay performed according to the manufacturer’s instructions. Trichostatin-A (TSA) was used as a positive control for HDAC inhibition [33]. Other controls included substrate, a blank (buffer only) and an untreated enzyme control. The% activity was calculated using the formula: (test sample RFU-blank RFU/untreated control RFU-blank RFU) x100) where RFU = relative fluorescence units.

PKC was measured from cell lysates of U1 cells treated with the complexes for 72 h (37°C, 5% CO_2_). Prostratin was used as a control for PKC activation. The PKC Kinase activity assay was obtained from Enzo Life Sciences (NY, USA) and performed according to the manufacturer’s instructions. Kinase activity was calculated using the formula: (Test compound absorbance–Blank absorbance)/(vehicle control absorbance-Blank absorbance) x 100.

#### Effect of complexes **1** and **2**on TNF-α production

The proinflammatory cytokine, TNF-α, stimulates the HIV-1 long terminal repeat (LTR) through the activation of NF-κB in both human CD4^+^ T cells and monocytes/macrophages [7]. Stimulants such as PMA have been reported to reactivate virus as a result of promoting the endogenous production of TNF-α which in turn stimulates the HIV-1 LTR. To determine if complexes **1** and **2** were associated with increases in TNF-α levels and thus viral reactivation, a cytometric bead array kit (CBA, BD BioSciences, California, USA) was used to assess the content of this cytokine in the U1 culture supernatants. The assay was performed according to the manufacturer’s instructions.

### Statistical analysis

Data analysis was performed using Graphpad Prism® (California USA) and a Student’s t test for unpaired observations. A p < 0.05 was considered significant. All data are presented as mean ± SEM where n = 3-6.

## Results and discussion

### U1 cell viability studies

**Figure 2:**
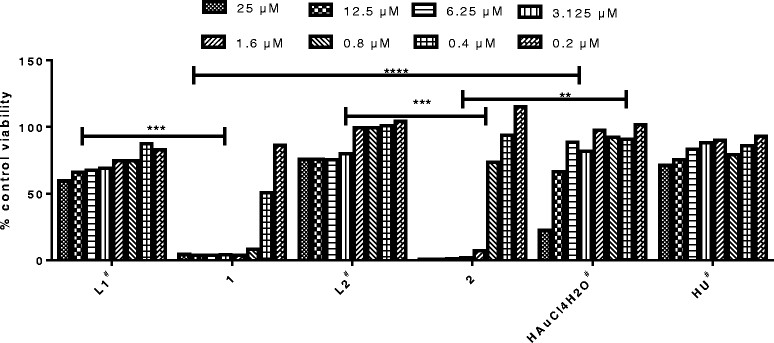
**Effect of complex 1 and 2 and the associated ligands on U1 cell viability.** A dose response curve was obtained for complexes 1 and 2 from 0.2-25 μM with CC_50_ values of 0.53 ± 0.12 and 1.00 ± 0.4 μM respectively. L1, L2 and HU were less toxic with CC_50_ > 100 μM while that of the gold (synthesis) starting material, HAuCl_4_.4H_2_O was >50 μM. The numbers above the bar graphs are the CC_50_ values. ^#^ Two fold serial dilutions from 100 down to 0.8 μM were performed. ****p < 0.0001, ***p = 0.0001, **p = 0.002.

A dose response curve from 0.2 – 25 μM of the viability of U1 cells was obtained for **1** and **2** and for the precursor ligands, HAuCl_4_.4H_2_O, and HU from 0.8-100 μM (Figure 2). The complexes were more toxic than the complementary ligands and gold starting material (p < 0.05) with 50% cytotoxic concentrations (CC_50_s) of 0.53 ± 0.12 μM and 1.00 ± 0.4 μM for **1** and **2** respectively. Ligands **L1** and **L2** as well as HU were non toxic with CC_50_ of > 100 μM while that of HAuCl_4_.4H_2_O was > 50 μM. Considering that toxic concentrations could provide false information on the effect of the complexes in the other cell-based assays, only non toxic concentrations were subsequently used. For complex **1**, concentrations of 0.2 μM and lower were used while for **2**, concentrations of 0.5 μM and lower were used since these resulted in more than 85% cell viability.

### Reactivation of latent HIV-1

**Figure 3:**
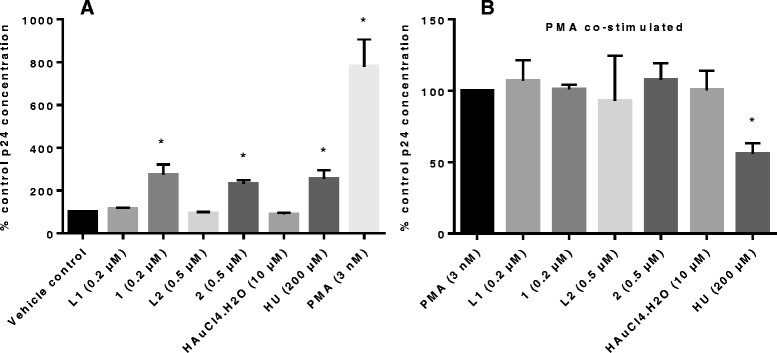
**Complex 1 and 2 reactivate HIV replication in latently infected U1 cells.** At non toxic concentrations of 0.2 and 0.5 μM viral p24 levels increased by 2.7 and 2.3 fold for complex 1 and 2 respectively (*p ≤ 0.03) while 200 μM HU, which was included in the study as a cytostatic positive control, reactivated latent virus by 2.6 fold **(A)**. PMA (3 nM), which was used both as a positive control for virus activation and to monitor the effect of the complexes on activated virus, significantly activated viral production (p = 0.01). HU also significantly (p = 0.01) inhibited PMA mediated latency activation by 44% when compared to PMA treated cells only, an observation which was absent for 1 and 2 **(B)**. The p24 levels for the vehicle control and PMA treated cells are represented as 100% so that differences resulting from complex effects could easily be differentiated.

For the activation studies, U1 cells were treated with complex only or co-stimulated with complex and PMA. Findings from these treatments are shown in Figure 3. To easily differentiate the effect of the complexes in each treatment type, the vehicle control and PMA treated cells were represented as 100% in Figure 3A and B respectively. For comparison of the differences between the vehicle control, complex treated and complex plus PMA, another representation of the data in Figure 3 is shown in Additional file 1: Figure S2 for complexes **1** and **2**.

#### In the absence of stimulant

The complexes were treated at three concentrations in a pre-screen (at CC_50_ or below, Additional file 1: Figure S1); complex **1** significantly reactivated viral production from the U1 cells by 2.7 fold (p = 0.023) when tested at 0.2 μM while complex **2** (0.5 μM), reactivated HIV replication by 2.3 fold (p = 0.005) when compared to untreated or cells only control (Figure 3A). At 200 μM, HU, a cytostatic agent used as a positive control, reactivated virus by 2.6 fold (p = 0.03). Complexes **1** and **2** were previously reported as cytostatic [24] and now with the ability to reactivate virus, have another activity in common with HU although demonstrating this activity at much lower concentrations. Ligands **L1** and **L2** had no effect on viral reactivation supporting our findings and previous reports for metal-based drugs that the metal entity is important for bioactivity. Also as expected, the gold starting material, HAuCl4.4H_2_O did not reactivate virus, further supporting the role of ligand complexation in metal-based drugs, as being activity enhancing [34]. The HIV-1 reactivation findings for these gold-based complexes are also supported by those reported for auranofin, another gold-based drug which induced HIV-1 reactivation from primary monocyte-derived macrophages as reported in the Additional file one by Shytaj *et al*. [35]. PMA reactivated virus production from the U1 cells (7.8 fold increase, Figure 3A) when compared to the vehicle control, confirming previous reports [12],[17].

#### PMA co-stimulation

In the co-stimulation treatment, the expectation was that the complexes would either synergistically reactivate virus in concert with PMA or inhibit virus production effected by the phorbol ester. For **1** and **2**, neither a synergistic reactivation nor inhibition of PMA mediated viral reactivation was observed at the tested concentrations since no significant increases or decreases in percentage reactivation was observed (Figure 3B and Additional file 1: Figure S2). Synergistic activation has been defined as the situation where the combination of two activators produces a level of activation that is greater than the sum of the effects produced with the individual activators [36]. This type of activation is important as it makes it possible to combine different latency activators to improve on the effectiveness of viral reactivation [18]. PMA’s activation of virus replication minimised the effects of all the treatments, except for that of HU which at 200 μM, inhibited p24 antigen production by 44% (p = 0.01, Figure 3B). This finding is not surprising since HU has been reported to inhibit HIV-1 LTR transactivation in the presence of both TNF-α and PMA as stimulants [19],[37]. According to Calzado and his colleagues [37], this inhibition of PMA mediated HIV-1 replication suggests that HU is capable of inhibiting HIV by another mechanism, in addition to RNR inhibition. HU inhibits activation mediated by PMA and TNF-α, which are both transcriptional activators but synergistically activates virus in the presence of the posttranslationally active cytokine, IL-6, by causing increases in Sp1 and Sp3 proteins which are involved in the expression of HIV-1 LTR [19],[38]. Considering that **1** and **2** did not inhibit viral production when co-stimulated with PMA, it is possible that the mechanism of activation is different from that of HU. Alternatively, it could be a concentration dependent issue and because higher concentrations of **1** and **2** were toxic to U1 cells, inhibition at higher concentrations would not be of value.

The reactivation for the complexes is shown here for the U1 promonocytic cell line model of latency as proof of concept that the cytostatic compounds also reactivate virus like HU. To have a better *in vivo* representation of the potential of these complexes as latency reactivators, a test on reactivation on T cells will be important since HIV predominantly resides in resting memory CD4+ T cells [39]. Unfortunately, another gold-based drug, auranofin was unable to activate latent HIV from primary CD4+ T cells [40]. The possibility that this could be applicable for complexes **1** and **2** which are also gold-based complexes exists. That notwithstanding, the complexes could play a role in reactivating virus from a subset of the immune system cells and further confirmation in a monocyte-derived macrophage cell line will be important in further exploring this potential.

### Viral reactivation mechanism

#### Complex effect on HDAC and PKC activity

**Figure 4:**
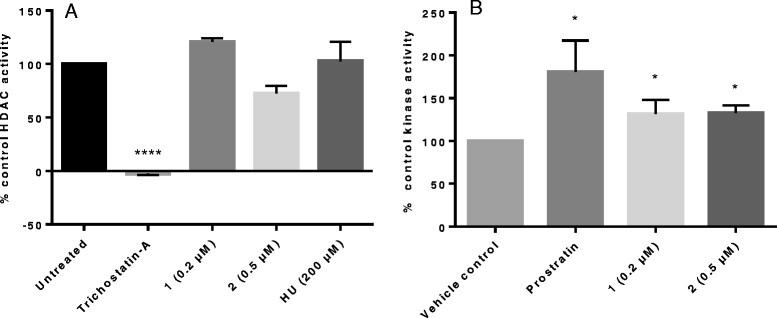
**The effects of the complexes on HDAC and PKC activity.** At a cut-off of ≥50% inhibition as inhibitory, complex 1 and 2 did not appreciably inhibit HDAC. The positive control, TSA, inhibited the enzyme by 103.7% with a p value of <0.0001 **(A)**. The enzyme control is represented as 100% inhibition. In the PKC assay, significant increases in PKC activity of 31.5 (p = 0.033) and 32.7% (p = 0.036) were observed for 1 and 2 respectively compared to the untreated vehicle control **(B)** suggesting that reactivation was as a result of an effect on NF-κB. Prostratin, a PKC activator caused significant increases in PKC of 80.3% (p = 0.02). The vehicle control sample is represented as 100% activity.

Because HDAC plays a role in maintaining latency, inhibitors of these enzymes reactivate latent virus. To determine whether complex **1** and **2**’s ability to reactivate virus was as a result of inhibiting HDAC, the activity of this enzyme in the presence of non-toxic concentrations of the complexes was evaluated using a fluorometric assay. Complexes **1** and **2** did not appreciably inhibit HDAC activity when a cut-off of 50% inhibition was considered. For **2**, a 27.4% inhibition was not significant (p > 0.05). TSA which was used as the positive control significantly (p < 0.0005) inhibited HDAC by 103.7% (Figure 4A).

Another mechanism by which viral latency activators function is by activating PKC. PKC activation by **1** and **2** significantly (p < 0.05) increased by 31.5 and 32.6% at 0.2 and 0.5 μM respectively compared to the vehicle only which was the 100% reference (Figure 4B). Kinase activity increased significantly by 80.3% for cells treated with 1 μg/mL of the positive control, prostratin.

These results indicate that PKC activation contributed to the HIV-1 reactivation observed for complexes **1** and **2** from the latently infected U1 cells. While complexes **1** and **2**, which are gold(III) thiosemicabazonate-based are structurally different from the known PKC activators, it is interesting to note that reactivation of HIV-1 is associated to the activation of this enzyme. Interestingly, in the co-stimulation studies with another PKC activator, PMA, no significant differences in viral reactivation was observed at the tested concentrations (Figure 3B and Additional file 1: Figure S2). A co-stimulation study with or without a PKC inhibitor should provide additional mechanistic information.

For the HDAC assay, the observed 27.4% inhibition for **2**, was not significant. The latter assay was not cell-based and higher concentrations of the complexes did not inhibit the enzyme (data not shown). Future studies to confirm the absence of HDAC activity will include doing the reactivation studies in the presence or absence of a histone acetyltransferases (HAT) inhibitor. Considering that the balance between protein acetylation and deacetylation controls several physiological and pathological cellular processes, the inhibition of HAT and thus alteration of the HAT/HDAC enzymes which maintain this balance [41] should provide additional mechanistic information. As part of continual investigations on the functionality of these complexes in HIV reactivation, these types of analysis will be considered.

#### Endogenous TNF-α production as viral reactivation mechanism

**Figure 5:**
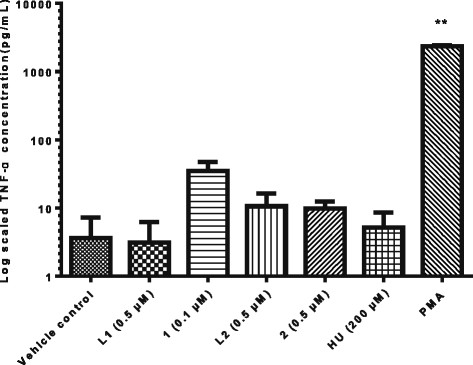
**Effect of complex 1 and 2 on TNF-α production from U1 cells.** Cell free supernatant from U1 cells treated with 1 and 2 was analysed using the CBA kit technology. TNF-α levels were increased by 9 and 3 fold for 1 and 2 respectively. The observed visual differences between treated and untreated cells was not statistically significant (p > 0.05) but could be contributing to the viral reactivation mechanism observed. PMA was used as a positive control and significantly stimulated TNF-α (p = 0.004). The data is plotted on a log scale, n = 3.

Stimulants such as PMA are associated with stimulating endogenous production of proinflammatory cytokines such as TNF-α [16], which in turn stimulates the HIV-1 LTR leading to viral reactivation. *In vitro* stimulation with TNF-α result in viral reactivation [11], further supporting this. TNF-α has been implicated in the immune disregulation observed in HIV since it promotes systemic inflammation resulting in disease progression *in vivo* [42] making it an important molecule in HIV infection. To further probe the mechanism by which complexes **1** and **2** reactivated virus, determination of TNF-α production was performed using the BD BioSciences (California, USA) CBA kit. The cytokines quantified by the kit included IL-2, IL-4, IL-6, IL-10, TNF-α, IFN-γ and IL-17A. For the purposes of this study, the main focus was on the endogenous production of TNF-α. TNF-α levels were shown to increase by 9 fold for complex **1** and 3 fold for complex **2** compared to the vehicle control (Figure 5) while for PMA treated cells, the increase was as a significant increase (p = 0.004) of up to 2353.86 ± 204 pg/mL (648 fold). Although increases were observed for **1**, the findings were not statistically significant (p > 0.05) suggesting that TNF-α stimulation might only be playing a contributory role which together with the increase in kinase activity resulted in HIV reactivation. Complex **1**’s effect on TNF-α was more elevated than **2** supporting the fact that reactivation of latent virus induced by **1** exceeded that of **2**.

At 200 μM, HU had no major effect on the production of TNF-α and because this compound also had no effect on PKC and HDAC, the mechanism by which HU reactivates virus probably differs from that of the complexes. In 1997, Navarra and his colleagues reported HU as capable of stimulating TNF-α production *in vivo* [43], and the lack thereof in this *in vitro* set up means other necessary factors required for an effect by HU on this cytokine, were absent. The cytostatic compound actinomycin D reactivates virus by modulating the cytokines IL-6 and TNF-β [20]. IL-6 levels were not affected by either the complexes or HU in our case. Complexes **1** and **2** also increased TNF-α levels in peripheral blood mononuclear cells from HIV negative donors (Additional file 1: Figure S3). This indicates that the *in vitro* upregulation of TNF-α occurs not only in the promonocytic U1 cell line but also *ex vivo* in primary immune system cells.

Upregulation of proinflammatory cytokine production by **1** and **2** (although not statistically significant) could imply that these complexes might be reactivating virus through a non specific mechanism, which is a concern with most latency activating agents [18]. Such a nonspecific mechanism might present these complexes as non selective meaning structural modifications for improved efficacy is needed. These modifications should also address the toxicity issues associated with the complexes with better targeting to latent reservoirs such as the use of nanotechnology [44].

Cytostatic agents arrest the cell cycle but according to Oguariri *et al*. [19], HU did not arrest U1 cells in the S and G2/M phases of the cell cycle as expected. This could be the reason why viral reactivation and hence replication occurred since retrovirus replication depends on cell cycling. Cell cycle analysis was performed for **1** and **2** and similarly to HU, cell cycle arrest of U1 cells was absent in the S and G2/M phases (data not shown).

## Conclusions

Inhibition of HIV by two bis(thiosemicabazonate) gold(III) complexes (**1** and **2**) was previously shown to be as a result of the cytostatic effects of the complexes as well as the lowering of CD4+ cell numbers [24], a finding also reported for HU [27]–[29]. Here we presented data indicating that the complexes had another characteristic that was similar to HU; viral reactivation potential, (with **1** being more potent than **2** possibly due to the ethyl group structural difference between the two). The mechanism by which **1** and **2** triggered viral reactivation in U1 cells was as a result of PKC activation (p < 0.05) with a minimal contribution from the endogenous production of TNF-α. Direct enzyme inhibition of HDAC was <50%. Considering that prostratin reactivates virus by activating PKC and at the same time upregulates TNF-α, the possibility that the complexes can do same exists. The increase in the endogenous production of TNF-α by viral reactivating agents like the phorbol esters, PMA and prostratin was previously reported [16],[17], and is confirmed here for PMA. The concern that most effective latency activators may cause generalised immune activation through the induction of abundant proinflammatory cytokines since the expression of HIV is closely linked to the activation state of the host cell [18], could be a concern for these complexes since this cytokine was minimally stimulated. Like prostratin, the combined effects executed means the complexes could eventually be recommended as inductive adjuvant therapy in HAART [17].

The reactivation of latent HIV-1 by these complexes are findings reported for the first time for metallodrugs that are also cytostatic (an indirect anti-viral property). Kinase enzyme activation and to an extent, endogenous TNF-α production were implicated in the reactivation mechanism. The results obtained support HIV-1 reactivation by the cytostatic complexes but further studies to determine the exact mechanism of reactivation of the dual (cytostatic and reactivation) role the complexes could play in viral “activation/elimination” is needed.

## Additional file

## Electronic supplementary material

Additional file 1: **This file contains a graph with data on the dose responsiveness of the reactivation effect caused by the complexes as Figure S1.** A figure representing data shown in Figure 3 of the main manuscript is presented in another format in **Figure S2.** while the effect of the complexes on endogenous production of TNF-α from PBMCs is included as **Figure S3.** (DOC 932 KB)

Below are the links to the authors’ original submitted files for images.

Authors’ original file for figure 1

Authors’ original file for figure 2

Authors’ original file for figure 3

Authors’ original file for figure 4

Authors’ original file for figure 5

## Abbreviations

CBA: Cytometric bead array
CC_50_: 50% cytotoxic concentration
dNTPs: deoxynucleotide triphosphates
HAART: Highly active antiretroviral therapy
HDAC: Histone deacetylases
HIV: Human immunodeficiency virus
HU: Hydroxyurea
IL: Interleukin
LTR: Long terminal repeat
MTT: 3-(4,5-dimethylthiazol-2-yl)-2,5-diphenyltetrazolium bromide
NF-κB: Nuclear Factor Kappa B
PBMCs: Peripheral blood mononuclear cells
PKC: Protein kinase C
PMA: Phorbol myristate acetate
RNR: Ribonucleotide reductase
TNF-α: Tumour Necrosis Factor alpha
