# Supplementary material for: In vitroreactivation of latent HIV-1 by cytostatic bis(thiosemicarbazonate) gold(III) complexes
Source: BMC Infect Dis. 2014 Dec 11;14:680. doi: 10.1186/s12879-014-0680-3 (PMC4265357; doi:10.1186/s12879-014-0680-3)
Supplement: Supplementary file 1 — Additional file 1: This file contains a graph with data on the dose responsiveness of the reactivation effect caused by the complexes as Figure S1. A figure representing data shown in Figure 3 of the main manuscript is presented in another format in Figure S2. while the effect of the complexes on endogenous production of TNF-α from PBMCs is included as Figure S3. (DOC 932 KB) [file 12879_2014_680_MOESM1_ESM.doc]

**Additional File 1_Fonteh and Meyer**

1. **Complex 1 and 2 reactivate virus from U1 cells in a dose dependent manner**

**Figure S1**: The effect of complexes **1** and **2** on p24 antigen production from U1 cells. Complexes **1** and **2** were tested at three concentrations of 0.1, 0.2 and 0.5 µM. At 0.5 µM of 1 (also the CC_50_), a decrease in p24 was observed probably due to toxicity while at 0.2 µM, a ~ 2 fold increase was observed. For complex **2**, a dose dependent increase in p24 production was observed at these concentrations. HU which was used as a positive control for viral reactivation increased p24 production by ~ 2.5 fold. This assay was performed as a pre-screen to show the dose response nature of the reactivation before subsequent assays for **1** and **2** at 0.2 and 0.5 µM. The p24 level for the untreated control (cells) was represented as 100% so that differences resulting from complex effects could easily be observed.

1. **Co-stimulation of complex 1 and 2 with PMA, a latency and PKC activator does not result in synergistic reactivation**

**Figure S2:** HIV-1 reactivation by complexes **1** and **2** and in combination with PMA. No significant differences in stimulation were observed for complexes co-stimulated with PMA compared to PMA stimulated cells only. It is possible that the effect of the complexes is minimised in the presence of PMA. The vehicle control is shown as 100% inhibition.

1. **Complex 1 and 2 endogenously cause the production of TNF-α from PBMCs**

**Figure S3:** Effect of complexes **1** and **2** on the endogenous production of TNF-α from PBMCs. The data was collected from PBMCs obtained from four HIV negative donors after written informed consent was obtained. Compared to the vehicle control, complexes **1** and **2** increased the production of TNF-α by ~ 3 fold each. Here, HAuCl_4_.4H_2_O appeared to be contributing to the increases in TNF-α observed for **1** and **2**. Such an increase was not observed for the precursor ligands **L1** and **L2**. At 100 µM, HU also did not alter TNF-α production.
