# Supplementary figures and images for: In vitroreactivation of latent HIV-1 by cytostatic bis(thiosemicarbazonate) gold(III) complexes
Source: BMC Infect Dis. 2014 Dec 11;14:680. doi: 10.1186/s12879-014-0680-3 (PMC4265357; doi:10.1186/s12879-014-0680-3)

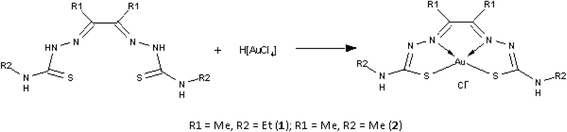

Supplement: Supplementary file 2 — Authors’ original file for figure 1 [file 12879_2014_680_MOESM2_ESM.gif]

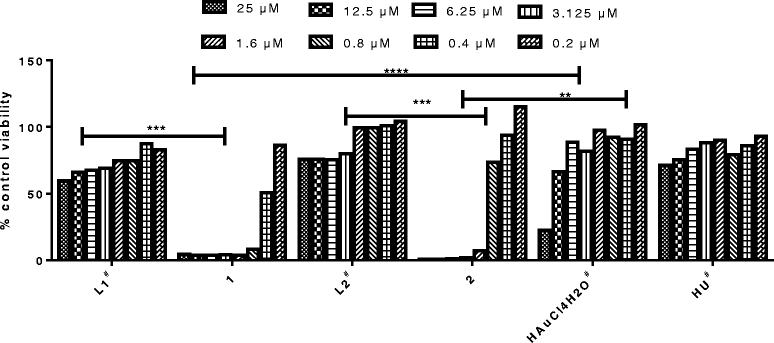

Supplement: Supplementary file 3 — Authors’ original file for figure 2 [file 12879_2014_680_MOESM3_ESM.gif]

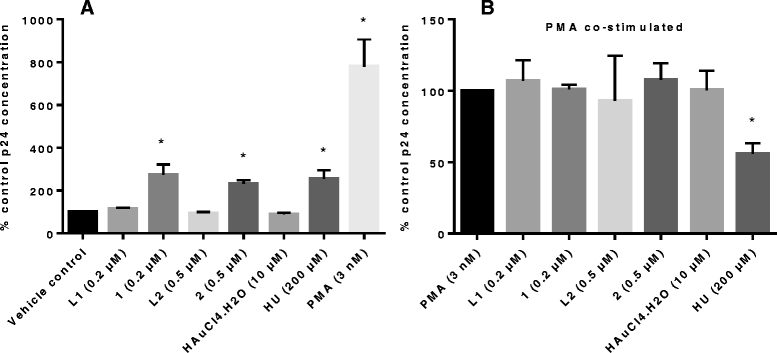

Supplement: Supplementary file 4 — Authors’ original file for figure 3 [file 12879_2014_680_MOESM4_ESM.gif]

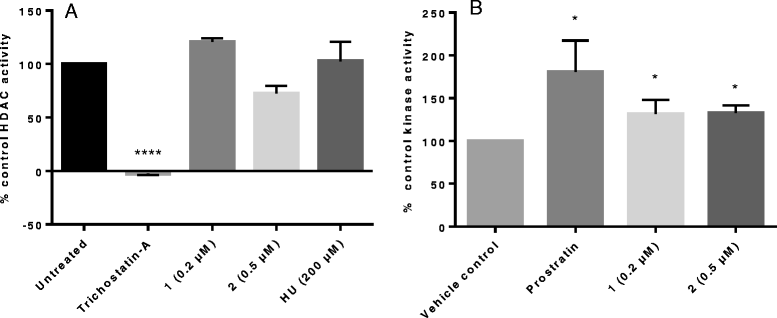

Supplement: Supplementary file 5 — Authors’ original file for figure 4 [file 12879_2014_680_MOESM5_ESM.gif]

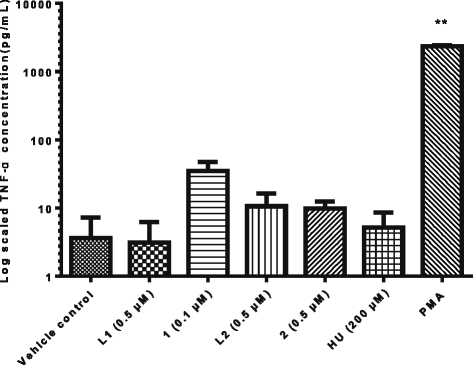

Supplement: Supplementary file 6 — Authors’ original file for figure 5 [file 12879_2014_680_MOESM6_ESM.gif]
